# NO_2_ inhalation induces maturation of pulmonary CD11c^+^ cells that promote antigenspecific CD4^+^ T cell polarization

**DOI:** 10.1186/1465-9921-11-102

**Published:** 2010-07-26

**Authors:** Samantha R Hodgkins, Jennifer L Ather, Sara A Paveglio, Jenna L Allard, Laurie A Whittaker LeClair, Benjamin T Suratt, Jonathan E Boyson, Matthew E Poynter

**Affiliations:** 1 The Vermont Lung Center and Department of Medicine, University of Vermont, Burlington, VT, 05405, USA; 2 Department of Surgery, University of Vermont, Burlington, VT, 05405, USA

## Abstract

**Background:**

Nitrogen dioxide (NO_2_) is an air pollutant associated with poor respiratory health, asthma exacerbation, and an increased likelihood of inhalational allergies. NO_2 _is also produced endogenously in the lung during acute inflammatory responses. NO_2 _can function as an adjuvant, allowing for allergic sensitization to an innocuous inhaled antigen and the generation of an antigen-specific Th2 immune response manifesting in an allergic asthma phenotype. As CD11c^+ ^antigen presenting cells are considered critical for naïve T cell activation, we investigated the role of CD11c^+ ^cells in NO_2_-promoted allergic sensitization.

**Methods:**

We systemically depleted CD11c^+ ^cells from transgenic mice expressing a simian diphtheria toxin (DT) receptor under of control of the CD11c promoter by administration of DT. Mice were then exposed to 15 ppm NO_2 _followed by aerosolized ovalbumin to promote allergic sensitization to ovalbumin and were studied after subsequent inhaled ovalbumin challenges for manifestation of allergic airway disease. In addition, pulmonary CD11c^+ ^cells from wildtype mice were studied after exposure to NO_2 _and ovalbumin for cellular phenotype by flow cytometry and *in vitro *cytokine production.

**Results:**

Transient depletion of CD11c^+ ^cells during sensitization attenuated airway eosinophilia during allergen challenge and reduced Th2 and Th17 cytokine production. Lung CD11c^+ ^cells from wildtype mice exhibited a significant increase in MHCII, CD40, and OX40L expression 2 hours following NO_2 _exposure. By 48 hours, CD11c^+^MHCII^+ ^DCs within the mediastinal lymph node (MLN) expressed maturation markers, including CD80, CD86, and OX40L. CD11c^+^CD11b^- ^and CD11c^+^CD11b^+ ^pulmonary cells exposed to NO_2 _*in vivo *increased uptake of antigen 2 hours post exposure, with increased ova-Alexa 647^+ ^CD11c^+^MHCII^+ ^DCs present in MLN from NO_2_-exposed mice by 48 hours. Co-cultures of ova-specific CD4^+ ^T cells from naïve mice and CD11c^+ ^pulmonary cells from NO_2_-exposed mice produced IL-1, IL-12p70, and IL-6 *in vitro *and augmented antigen-induced IL-5 production.

**Conclusions:**

CD11c^+ ^cells are critical for NO_2_-promoted allergic sensitization. NO_2 _exposure causes pulmonary CD11c^+ ^cells to acquire a phenotype capable of increased antigen uptake, migration to the draining lymph node, expression of MHCII and co-stimulatory molecules required to activate naïve T cells, and secretion of polarizing cytokines to shape a Th2/Th17 response.

## Background

The prevalence of allergic asthma has risen steadily in recent decades, making the disease a primary public health concern [1]. Potential explanations for the increase include reduced exposure to infectious agents during childhood, dietary changes, and exposure to environmental pollutants. Allergic asthma is caused primarily by an inappropriate CD4^+ ^Th2 response, which results in symptoms mediated by Th2 cytokines, including IL-13 provoking airways hyperresponsiveness and mucus production, IL-4 promoting the production of antigen specific IgE, and IL-5 inducing eosinophilia [2]. Recent evidence suggests that Th17 cells secreting IL-17 are associated with a severe [3], steroid-resistant [4] form of allergic asthma. However, the underlying mechanisms that initiate the aberrant T cell response in allergic asthma are still not well understood (reviewed in [5]). Our lab has shown that inhalation of the gaseous air pollutant and endogenously-generated reactant nitrogen dioxide (NO_2_) is capable of acting as an adjuvant, promoting allergic sensitization to the innocuous protein ovalbumin (ova) in a novel mouse model [6]. This model is physiologically relevant as antigen sensitization occurs via inhalation, as would typically occur in humans and does not require an additional adjuvant [7]. NO_2 _has also been correlated with poor respiratory health [8], exacerbating existing asthma in animal models [9] and in human subjects [10], as well as with an increased likelihood of inhalational allergies [11] and developing asthma in human studies [12].

Pulmonary antigen-presenting cells, especially dendritic cells (DCs), express the surface marker CD11c [13] and have a potent ability to induce the proliferation and activation of naïve T cells and to secrete inflammatory and T-helper cell polarizing cytokines [14-16]. CD11c^+ ^cells are critical for initiating and shaping the antigen-specific adaptive immune response and are critical during the reactivation of CD4^+ ^T cells *in vivo *[17]. CD11c^+ ^DCs are capable of these activities because they possess multiple unique characteristics. First, DCs are strategically located beneath the airway epithelium and continually take up antigen under steady-state conditions [15]. Second, DCs can undergo maturation upon exposure to inflammatory stimuli and travel to draining lymph nodes, presenting antigens in the context of both MHCI and MHCII. Finally, DCs express co-stimulatory molecules and secrete polarizing cytokines necessary to initiate and shape the T cell mediated immune response [16,18]. However, defining DCs via surface marker expression remains complicated, especially in non-lymphoid tissues such as the lung, due to the number of different methods described in the literature and the shared cell surface markers expressed by several cell subsets. The myeloid DC subset is attributed with T cell stimulatory capacity, having the ability to induce Th1, Th2, or Th17 type responses [19], as well as non-inflammatory T regulatory (Treg) responses [20]. Myeloid DCs in the lung have been defined as CD11c^+^CD11b^+ ^[19,21], CD11c^+^MHCII^+ ^[22], or CD11c^+ ^alone or in combination with low FITC auto-fluorescence [23,24]. This variation is further complicated by the overlap of markers with multiple other cell types, the most prominent of which in the lung is CD11c^+ ^macrophages [21]. Plasmacytoid DCs (pDCs) are also present within the lung [25] and have been shown to exert an anti-inflammatory role, decreasing both the ability of mDCs to generate effector T cells as well as inducing Treg cell proliferation [26]. This pDC population expresses B220 (CD45RB) as well as low levels of Gr-1, making them more difficult to delineate from B cells and granulocytes than myeloid DCs [18]. Recently, another population of dendritic cells called inflammatory DCs has been described (CD11b^+^Gr-1^lo^F4/80^lo^), which traffic from the blood to sites of inflammation, upregulate CD11c upon arrival in the tissue, and acquire the DC characteristics of migration to the draining lymph node and induction of T cell proliferation [22,27].

DCs are capable of skewing the T helper cell response through their ability to express distinct patterns of co-stimulatory molecules as well as to produce cytokines that create an environment for differential T cell polarization [19]. Expression of the co-stimulatory molecules CD86 and OX40L have been shown to promote naïve CD4^+ ^T cells to develop a Th2 phenotype [19,28,29]. Importantly, OX40L-deficient mice are protected from allergic sensitization and Th2 mediated inflammation, indicating that OX40L plays a critical role in the generation of Th2 immune responses [29]. DCs also regulate Th2 cell differentiation and expansion by producing IL-6 [30,31]. Th17 cells may also be induced by DC production of IL-6 in combination with TGFβ or IL-23 [30,32,33], or by IL-1β alone [34], while IL-12 alone promotes a Th1 response [35]. Thus, activated DCs as a cellular source of co-stimulatory molecules and polarizing cytokines function as important regulators of CD4^+ ^mediated T cell responses.

Since activation of pathogenic CD4^+ ^Th2 cells is believed to be dependent on CD11c^+ ^DCs [36], we tested whether CD11c^+ ^cells are necessary for NO_2_-promoted allergic sensitization. We used transgenic mice in which CD11c^+ ^cells can be temporarily depleted due to the incorporation of a transgene encoding a simian Diphtheria Toxin Receptor (DTR) and Green Fluorescent Protein (GFP) fusion protein under the control of the murine CD11c promoter (CD11c-DTR mice) [37,38]. As murine cells lack the DT receptor [17] and only CD11c^+ ^cells in CD11c-DTR Tg^+ ^mice express the DT receptor, administration of DT to these mice results in inducible ablation of 90% of CD11c^+ ^cells within the mouse lasting for approximately 48 hours [38]. Using CD11c-DTR mice, it has been demonstrated in models of allergic asthma that CD11c^+ ^DCs are important during allergic sensitization induced by the i.p. injection of ova in combination with the adjuvant Alum [22] and are also critical during allergen challenge [17]. Importantly, the use of these mice to assess the role of CD11c^+ ^cells during allergic sensitization via inhalation, and in response to NO_2_, is so far unpublished.

In these studies, we demonstrate that when CD11c^+ ^cells were depleted during sensitization, mice exhibited less inflammation within the lung following allergen challenge and displayed a significantly impaired capacity for CD4^+ ^T cells to secrete Th2 and Th17 cytokines *in vitro*, implying that CD11c^+ ^cells are critical during NO_2_-promoted allergic sensitization. Furthermore, flow cytometric analyses of CD11c^+ ^cell populations within the lung and draining lymph node revealed that NO_2 _increased MHCII and costimulatory molecule expression in a temporally-orchestrated fashion. Finally, CD11c^+ ^pulmonary cells exposed to NO_2 _increased antigen capture of ova-Alexa 647, produced significant amounts of IL-1α, IL-1β, IL-12p70, and IL-6 compared to air controls, and augmented the production of IL-5 by CD4^+ ^T cells *in vitro*. These results suggest a critical function for pulmonary CD11c^+ ^cells during NO_2_-promoted allergic sensitization, including augmented pulmonary CD11c^+ ^cell recruitment and an improved ability to uptake and present antigen, provide co-stimulation, and secrete polarizing cytokines that initiate and shape a Th2/Th17-biased immune response. Comprehending the mechanisms underlying NO_2_-promoted allergic sensitization provides insight into the potential for common environmental pollutants to affect the development of increasingly prevalent pulmonary disorders, such as allergic asthma.

## Methods

### Mice

C57BL/6 mice were purchased from the Jackson Laboratory and used in experiments and as antigen presenting cell donors. OTII TCR transgenic mice on the C57BL/6 background were purchased from the Jackson Laboratory and bred at the University of Vermont as a source of CD4 T cells responsive to the peptide ova_323-339_, an immunodominant MHCII antigenic epitope from the protein ovalbumin [39]. CD11c-DTR-EGFP (CD11c-DTR) transgenic mice were purchased from Jackson Laboratories (Bar Harbor, ME) (B6.FVB-Tg(Itgax-DTR/EGFP)57Lan/J). The CD11c-DTR mice were generated with a transgene insert containing the Itgax (CD11c) promoter driving expression of a fusion protein containing the diphtheria toxin receptor (DTR) and the enhanced green fluorescent protein (GFP). The CD11c-DTR transgenic and transgene-negative littermates used in these studies were bred and raised at the University of Vermont. Eight- to fifteen-week old female mice, weighing approximately 20-25 g, were used for all studies. Mice were euthanized by a lethal dose (150 mg/kg) of pentobarbital (Nembutal, Ovation Pharmaceuticals Inc., Deerfield, IL) via intraperitoneal (i.p.) injection. All experiments were conducted following the guidelines and under the approval of the University of Vermont's Institutional Animal Care and Use Committee. The mice were housed in a facility accredited by the American Association for the Accreditation of Laboratory Animal Care.

### In vivo NO_2 _and ovalbumin exposures

1000 ppm nitrogen dioxide with a nitrogen balance (AirGas, Salem, NH) was diluted to 15 ppm with HEPA-filtered room air in a specially designed glass chamber (Specialty Glass, Inc., Rosharon, TX) within a fume hood. As needed, control mice were exposed to HEPA-filtered room air in an identical chamber for the same duration as NO_2_-exposed mice. A calibrated nitric oxide analyzer, equipped with an NO_2 _thermal converter, was used to measure NO_2 _in the gas phase, according to manufacturer's instructions (EcoPhysics, Ann Arbor, MI). For studies in which exposure to ovalbumin was performed, mice were exposed to aerosolized 3.4% ovalbumin (final concentration 10 mg/ml) (Grade V, Sigma, St. Louis, MO) in Dulbecco's Phosphate-Buffered Saline (DPBS) (CellGro, Manassas, VA) for 30 minutes immediately following exposure to either NO_2 _or air or during antigen challenge. The contaminating endotoxin concentration in the ovalbumin preparation used for inhalation was measured to be 1.5 ng/mg protein using a chromogenic Limulus assay (GenScript, Piscataway, NJ).

### NO_2_-promoted allergic sensitization model

Eight-week-old C57BL/6 female mice or eight to fifteen-week-old male and female CD11c-DTR mice (Tg^+ ^and Tg^-^) were exposed to 15 ppm NO_2 _for 1 hour followed by 30 minutes of aerosolized ova on day 0. This acute pro-inflammatory concentration of NO_2 _was established in a separate dose response study to be the minimum sufficient to promote allergic sensitization using this regimen. Mice were then challenged with aerosolized ova on days 14, 15, and 16 and analyzed 48 hours later (day 18). As previously described [6], mice exposed to NO_2 _and saline during the sensitization phase do not elicit allergic responses and were only included in limited numbers as controls to assure that in wildtype mice the NO_2_/ova exposure regimen induced airway eosinophilia, elevated levels of antigen-specific immunoglobulins in the serum, and antigen-specific cytokine production during restimulation of CD4^+ ^T cells *in vitro *compared to these controls.

### Administration of diphtheria toxin

As dictated by the experiment, Tg^+ ^and Tg^- ^CD11c-DTR mice were administered Diphtheria toxin (DT) (Sigma, St. Louis, MO) or saline via i.p. injection 24 hours prior to either sensitization or flow cytometric analyses for depletion of CD11c^+ ^cells. A dose of 4 ng/g of body weight was used at a concentration of 0.2 or 0.25 ng/μl.

### Bronchoalveolar lavage (BAL)

Following euthanasia, BAL was collected by instilling and recovering 1 ml of DPBS (CellGro, Manassas, VA) containing protease inhibitor cocktail (Sigma, St. Louis, MO) into the lung via a tracheal cannula. Total and differential cell counts were performed using the Advia 120 automated hematology analyzer system and cytospins. For the cytospins, cells were centrifuged onto glass slides at 800 rpm (RCF = 80 × *g*) and stained using the Hema3 kit (Biochemical Sciences, Inc., Swedesboro, NJ), with differential cell counts performed on at least 200 cells [40]. BALF supernatants were collected for protein quantitation by Bradford assay (Bio-Rad, Hercules, CA) and cytokine quantitation by Bio-Plex (Bio-Rad).

### Ova-specific IgE and IgG_1 _quantification

Following euthanasia, approximately 300 μl of blood was collected via cardiac puncture of the right ventricle using a 26-gauge needle attached to a 1 ml syringe into serum separator tubes (BD Biosciences, San Diego, CA). The blood was centrifuged at 13,200 rpm and serum was stored at -80°C. Ova-specific immunoglobulins were quantified utilizing a two-step sandwich (capture) ELISA. 96-well high-binding plates (Costar, Bethesda, MD) were coated with 2 μg/ml anti-mouse IgE (BD Pharmingen clone R35-72) or IgG_1 _(BD Pharmingen clone A85-3) mAb in DPBS for 1 hour at RT. Plates were washed with 0.05% Tween 20 (Fisher Scientific, Pittsburgh, PA) in PBS, blocked for 1 hour with PBS/1% BSA (Fischer Scientific), and serum samples were diluted (1:10 for IgE, 1:1000 for IgG_1_) and added in duplicate in PBS/1% BSA for 1 hour at RT. Plates were washed and incubated with a 1:2500 dilution of digoxigenin-coupled ova (Roche, Madison, WI) in PBS/1% BSA for 1 hour at RT. Plates were washed and incubated with a 1:2000 dilution of anti-digoxigenin-Fab coupled to peroxidase (Roche) in PBS/1% BSA for 30 minutes. Plates were washed, developed using reagents from R&D Systems (Minneapolis, MN), stopped with 1 N H_2_SO_4_, and ODs were read using a Bio-Tek Instruments PowerWave_X _at 450 nm with background subtraction at 570 nm.

### CD4^+ ^T cell re-stimulation and cytokine analyses

Single-cell suspensions were generated from spleens and mediastinal lymph nodes (MLNs) by passing the tissues through a 70 μm nylon mesh filter (BD Biosciences) and lymphocytes were enriched by separation with Lymphocyte Separation Medium (MP Biomedicals, Irvine, CA). CD4^+ ^T cells were isolated by positive selection using CD4 magnetic microbeads (Miltenyi Biotec, Bergisch Gladbach, Germany), according to the manufacturer's instructions. CD4^+ ^T cells (2 × 10^6 ^cells/ml) were stimulated with 100 μg/ml ova in the presence of C57BL/6 antigen presenting cells (APCs) (4 × 10^6 ^cells/ml). APCs were obtained by splenic T cell depletion by negative selection using Abs to CD4, CD8, and Thy-1, and treatment with rabbit complement and mitomycin C, as previously described [41]. Following 96 hours of stimulation, supernatants were collected and analyzed by ELISA using reagents and instructions from R&D Systems. ODs from duplicate samples and duplicate standards were read using a Bio-Tek Instruments PowerWave_X _at 450 nm with background subtraction at 570 nm.

### Analysis of CD11c^+ ^cells from the lung and mediastinal lymph node (MLN)

The lung was dissociated to a single cell suspension by mechanical disruption followed by incubation at 37°C with 0.5 mg/ml Liberase Blendzyme 3 (Roche Applied Science, Indianapolis, IN) and 40 μg/ml DNase for a total of 40 minutes. Red blood cells were lysed using 0.83% ammonium chloride and the lung suspension was filtered through a 40 μm nylon mesh membrane (BD Biosciences, San Diego, CA). Total cells were then counted using Advia or hemocytometer and were stained, as described below, or used for the isolation of CD11c^+ ^pulmonary DCs via positive selection using MACS columns (Miltenyi Biotec, Bergisch Gladbach, Germany) for *in vitro *studies, according to the manufacturer's instructions. MLN cells were dissociated by mechanical disruption, filtered through a 40 μm nylon mesh membrane (BD Biosciences), and stained as described below.

### Cell staining and fluorescence-activated cell scanning (FACScan)

Lung and MLN derived cells were stained with the following antibodies: CD45-PO, CD11c-PETR, F4/80-Alexa 647, CD86-Alexa 647 (all from Caltag, Carlsbad, CA); I-A/I-E-PerCP/Cy5.5 (BioLegend, San Diego, CA); CD11b-APCcy7 and GR-1-PE (all from BD Pharmingen, San Diego, CA). To assess maturation, cells were also stained for CD80-PE, CD40-APC, and biotinylated OX40L (all from BD Pharmingen). Biotinylated antibodies were detected using strepavidin-PE (BD Pharmingen). 1 × 10^6 ^cells were first blocked with 2.5 μg/ml Fc block (anti-CD16/CD32, BD Pharmingen) for 30 minutes, washed in FACS buffer (DPBS (CellGro) with 5% FBS (Gibco, Carlsbad, CA)), and then stained for 30 minutes in 100 μl of antibody solution at the optimal concentration. In the case of a biotinylated primary antibody, cells were washed and stained with the secondary antibody for 30 minutes. Following staining, all cells were washed and fixed in DPBS with 5% FBS and 1% paraformaldhehyde. Cells were analyzed on the flow cytometer 1-3 days following staining using a Becton Dickinson LSR II FACS equipped to distinguish as many as 7 fluorophores. Dead cells were excluded from analysis by FSC and SSC gating.

### Oropharyngeal aspiration of ova-Alexa 647

Following exposure to 1 hour of air or 15 ppm NO_2_, mice were anesthetized with inhaled isoflurane (Webster Veterinary, Sterling, MA) and administered 50 μl of 1 μg/μl ova-Alexa 647 (Invitrogen, Carlsbad, California) via oropharyngeal aspiration [42]. Lungs and MLNs were taken 2 and 48 hours post exposure and processed into single cell suspensions for flow analysis of CD11c^+ ^cells and uptake of ova-Alexa 647.

### Ex vivo co-culture of CD11c^+ ^pulmonary cells and CD4^+ ^T cells

CD11c^+ ^cells from the lung (2 × 10^6 ^cells/ml) were co-cultured with OTII CD4^+ ^T cells (1 × 10^6 ^cells/ml) and 100 μg/ml of ova_323-339 _in a total volume of 200 μl/well in a flat-bottomed 96-well tissue culture plate. After 96 hours, cell-free supernatants were collected and frozen at -20°C. Cell-free supernatants were analyzed for the following mediators: IL-2, IL-3, IL-4, IL-5, IL-6, IL-9, IL-10, IL-12p40, IL-12p70, IL-13, IL-17, IFNγ, IL-1α, IL-1β, G-CSF, GM-CSF, KC, MCP-1, MIP-1α, MIP-1β, TNFα, RANTES, and Eotaxin using Bio-Plex (Bio-Rad).

### Statistical analyses

Data were analyzed by two-tailed unpaired Student's *t *test or by two-way ANOVA with Bonferroni post test. Statistical calculations were performed using GraphPad Prism 5 for Windows. *p *values smaller than 0.05 were considered statistically significant.

## Results

### Depletion of CD11c^+ ^cells prevents NO_2_-promoted allergic sensitization and manifestation of an allergic asthma phenotype

**Figure 1:**
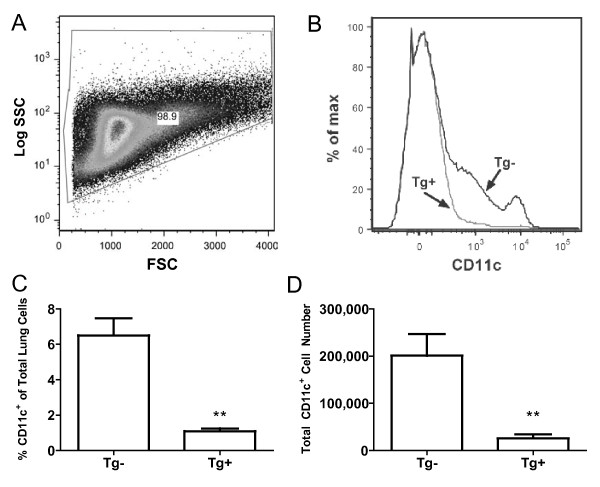
**Diphtheria toxin-mediated depletion of CD11c**^**+ **^**cells in the lung**. Eight- to fifteen-week-old female CD11c-DTR Tg^+ ^and Tg^- ^mice were administered 4ng DT/g of body weight via i.p. injection. Twenty-four hours later, lungs were harvested, digested into single-cell suspensions, and immunostained. Total lung cells were visualized by flow cytometry (A) and CD11c^+ ^cells from the parent gate were identified (B). Graphs show percent CD11c^+ ^cells from the lung (C) and total number of CD11c^+ ^cells in the lung, calculated based on the % CD11c^+ ^cells multiplied by the number of cells in the single-cell suspension from the lungs (D). Values are mean ± SEM with 4 animals per group. ** denotes *p *< 0.01 by Student's t test.

**Figure 2:**
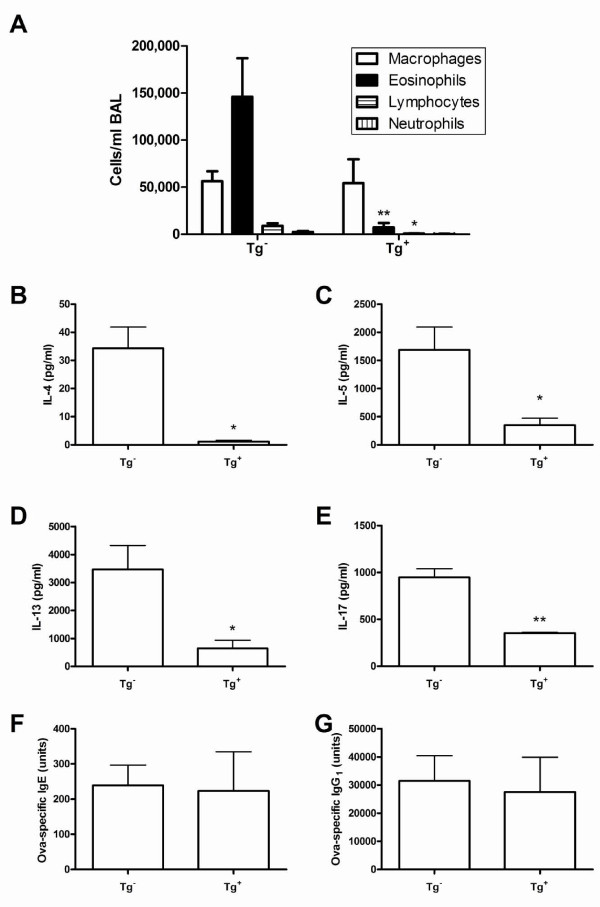
**Effects of depleting CD11c**^**+ **^**cells during sensitization in an NO**_**2**_**-promoted allergic asthma model**. Eight- to fifteen-week-old female CD11c-DTR Tg^+ ^and Tg^- ^mice were administered 4 ng DT/g of body weight via i.p. injection on day -1. All mice then underwent inhalation of 15 ppm NO_2 _for 1 hour followed by 30 minutes of aerosolized ova on day 0. All mice were challenged with aerosolized ova on days 14, 15, and 16. Differential cell counts were measured from the BAL fluid 48 hours after the final ova challenge (A). Values shown are mean ± SEM with 8 Tg- and 5 Tg+ mice per group. CD4^+ ^cells were isolated from spleens by positive selection on day 18 and co-cultured with antigen presenting cells (APCs) from naïve C57BL/6J mice and 100 μg/ml ova. Conditioned medium was collected at 96 hours and analyzed for the Th2 cytokines IL-4 (B), IL-5 (C), and IL-13 (D), and the Th17 cytokine IL-17 (E) by ELISA. No cytokines were detected in medium from APCs cultured alone or from CD4^+ ^T cells cultured with APCs in the absence of ova. Values shown are mean ± SEM with 4-5 mice per group. The ova-specific immunoglobulins IgE (F) and IgG_1 _(G) were measured from serum collected 48 hours after the final ova challenge (day 18) by ELISA using serum from Alum/ova-immunized mice to generate standard curves. Values shown are mean ± SEM with 7-10 mice per group. Statistics were computed by unpaired Student's t test. * denotes *p *< 0.05 and ** denotes *p *< 0.01 compared with respective Tg- samples.

Optimal depletion of CD11c^+ ^cells in the spleen of CD11c-DTR mice has previously been reported to occur between 24 and 48 hours post i.p. administration of DT, with CD11c^+ ^cells beginning to re-populate by 72 hours [38]. To verify that depletion of CD11c^+ ^cells within the lung and MLN could be achieved in Tg^+ ^animals following intraperitoneal injection of DT, Tg^- ^and Tg^+ ^mice were administered DT and then analyzed 24 hours later. Single-cell suspensions from the lung were stained for CD11c and analyzed by flow cytometry. CD11c^+ ^cells in the lung (Figure 1) were substantially depleted 24 hours post administration of DT in CD11c-DTR Tg^+ ^mice. We next set out to test whether CD11c^+ ^cells were necessary during NO_2_-promoted allergic sensitization, and thus if depletion of these cells during sensitization would diminish features of allergic asthma in mice. We adjusted our previously-published model [6] to require only one sensitization, and thus only one administration of DT. Tg^- ^and Tg^+ ^mice were administered DT via i.p. injection on day -1 and underwent inhalation of 15 ppm NO_2 _for 1 hour followed by 30 minutes of aerosolized ova on day 0. Mice were then challenged with aerosolized ova on days 14, 15, and 16 and analyzed 48 hours later (day 18). Tg^- ^mice exhibited features of allergic asthma, including elevated cell counts and eosinophils in BAL fluid, increased ova-specific IgE and IgG_1 _in the serum, and secretion of Th2 and Th17 cytokines upon re-stimulation of CD4^+ ^T cells *in vitro*. Compared with Tg^- ^mice, Tg^+ ^animals displayed significant decreases in the numbers of eosinophils and lymphocytes within the BAL (Figure 2A). In addition, when CD4^+ ^T cells were restimulated with APCs and ova, there were significant decreases in the levels of IL-4, IL-5, IL-13, and IL-17 (Figure 2B-E). Despite these decrease in cytokine production, there were no reductions in the serum levels of ova-specific IgE or IgG_1 _(Figure 2F-G).

### NO_2 _and ova exposure increases the number and maturation status of CD11c^+^CD11b^- ^cells within the lung

**Figure 3:**
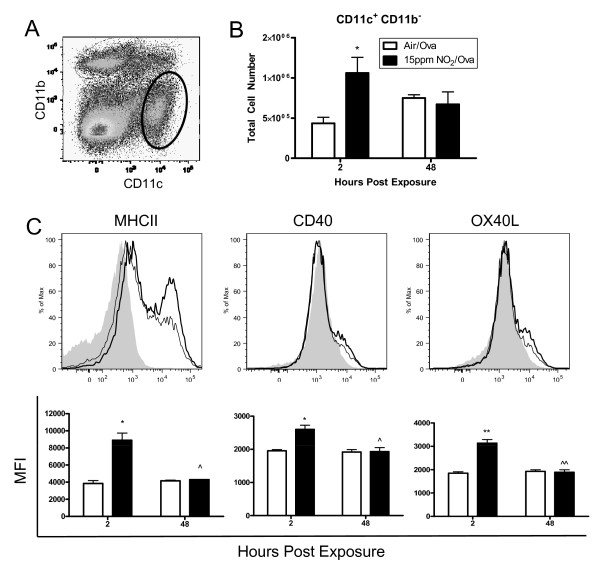
**Number and maturation status of lung CD11c**^**+**^**CD11b**^- ^**cells following NO**_**2 **_**and ova exposure**. Mice were exposed to 1 hour of 15ppm NO_2 _or air followed by 30 minutes of aerosolized 3.4% ova. Lungs were harvested 2 and 48 hours later, single cell suspensions were generated and stained with antibodies, and cells were analyzed by flow cytometry. Dead cells were excluded by FSC and SSC gating and then total lung cells were gated comparing CD11c and CD11b expression, with future analyses focusing on the CD11c^+^CD11b^- ^population (bold gate, A). The total number of CD11c^+^CD11b^- ^cells within the lung was enumerated (B) and the maturation status of these cells was assessed by median fluorescence intensity (MFI) of MHCII and the co-stimulatory molecules CD40 and OX40L 2 hours post exposure (C). NO_2_-exposed animals are represented by a thick line or black bar while air-exposed animals are represented by a thin line or white bar. Grey, filled histograms are isotype controls. Data shown are mean ± SEM with 3 animals per group and are representative of experiments performed twice. * denotes *p *< 0.05 and ** denotes *p *< 0.01 versus air control at the same time point by unpaired Student's t test. ^ denotes p < 0.05 and ^^ denotes *p *< 0.01 versus 2 hours post NO_2 _exposure by unpaired Student's t test.

To understand immunological changes in the lung following NO_2 _exposure that could allow for allergic sensitization in our mouse model [6], we investigated if the CD11c^+ ^cell populations within the lung were increased or activated following NO_2_/ova exposure. C57BL/6 mice were exposed to 1 hour of 15 ppm NO_2 _followed by 30 minutes of aerosolized 3.4% ova and the lung and MLN were digested and analyzed by flow cytometry at 2 and 48 hours. Compared to air/ova exposed mice, bronchoalveolar lavage fluid recovered at 2 hours from the NO_2_/ova-exposed mice contained elevated levels of total protein (239.5 vs. 70.8 μg/ml; p = 0.040), IL-6 (315.8 vs. 157.5 pg/ml; p = 0.009), and MCP-1 (689.3 vs. 143.4 pg/ml; p = 0.026), indicative of the effects of 1 hour of 15 ppm NO_2 _exposure, which manifest in a degree of lung damage and cytokine production similar to what we have previously reported [6]. Since pulmonary DCs are defined by a variety of cell surface markers, including primarily CD11c, CD11b, and MHCII [19,22,23], we assessed the number and maturation status within two separate cell populations that potentially contain DCs. These include CD11c^+^CD11b^- ^cells and CD11c^+^CD11b^+ ^cells. While inflammatory CD11b^+^Gr-1^lo^F4/80^lo ^DCs [22,27] were also studied, no significant alterations in this population were found and these data are therefore not presented. 2 hours post NO_2_/ova exposure, the number of CD11c^+^CD11b^- ^cells within the lung (Figure 3A) nearly doubled compared to the air/ova control (Figure 3B). Importantly, NO_2_-exposed CD11c^+^CD11b^- ^cells expressed higher levels of MHCII, CD40, and OX40L (Figure 3C), indicating that resident CD11c^+ ^cells matured or that newly recruited CD11c^+ ^cells were of a more mature phenotype. These CD11c^+^CD11b^- ^cells from air/ova- vs. NO_2_/ova-exposed lungs at 2 hours were on average 29.0 ± 1.5% vs. 42.4 ± 0.6% (p ≤ 0.01) MHCII^+^, 11.4 ± 0.09% vs. 16.3 ± 0.8% (p ≤ 0.05) CD40^+^, and 13.6 ± 1.2% vs. 16.3 ± 0.8% (p ≤ 0.05) OX40L^+^, differences that returned to baseline levels by 48 hours. CD11c^+ ^DCs expressing MHCII and CD40 have been shown to have a potent ability to cause T cell proliferation [16], while DCs expressing OX40L have been shown to induce naïve T cells to become Th2 polarized [43]. Thus, these changes in the CD11c^+^CD11b^- ^cell population in the lung may have an important role in NO_2_-promoted allergic sensitization.

**Figure 4:**
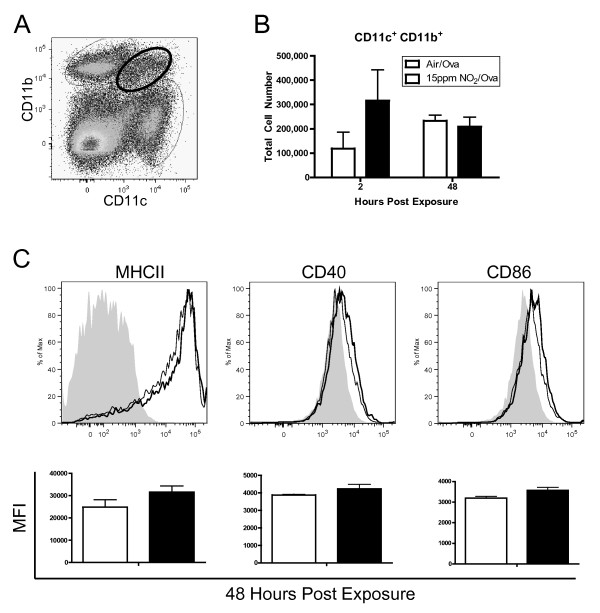
**Number and maturation status of lung CD11c**^**+**^**CD11b**^**+ **^**cells following NO**_**2 **_**and ova exposure**. Mice were exposed to 1 hour of 15 ppm NO_2 _or Air followed by 30 minutes of aerosolized 3.4% ova. Lungs were harvested 2 and 48 hours later, single cell suspensions were generated and stained with antibodies, and cells were analyzed by flow cytometry. Dead cells were excluded by FSC and SSC gating and then total lung cells were gated comparing CD11c and CD11b expression, with future analyses focusing on the CD11c^+^CD11b^+ ^population (bold gate, A). The total number of CD11c^+^CD11b^+ ^cells within the lung was enumerated (B) and the maturation status of these cells was assessed by expression of MHCII and the co-stimulatory molecules CD40 and CD86 48 hours post exposure (C). Maturation markers are graphed as median fluorescence intensity (MFI). NO_2_-exposed animals are represented by a thick line or black bar while air-exposed animals are represented by a thin line or white bar. Grey, filled histograms are isotype controls. Data shown are mean ± SEM with 3 animals per group and are representative of experiments performed twice. Differences did not reach statistical significance by unpaired Student's t test.

**Figure 5:**
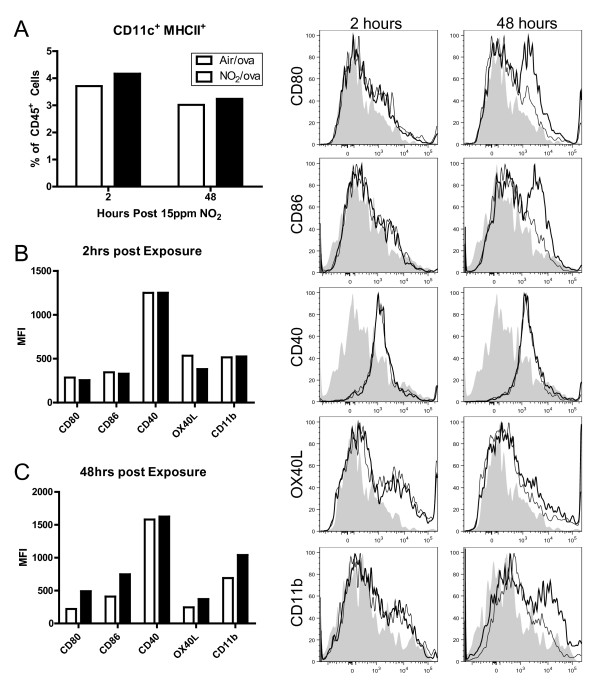
**CD11c**^**+ **^**dendritic cell maturation status in mediastinal lymph nodes (MLN) following NO**_**2 **_**and ova exposure**. Mice were exposed to 1 hour of 15 ppm NO_2 _or air followed by 30 minutes of aerosolized 3.4% ova. Lungs were harvested 2 and 48 hours later, single cell suspensions were generated and stained with antibodies, and cells were analyzed by flow cytometry. Only CD45^+ ^MLN cells were included in analyses. NO_2_-exposed animals are represented by a thick line while air-exposed animals are represented by a thin line. Grey, filled histograms are isotype controls. CD11c^+^MHCII^+ ^cells were measured as a percent of all CD45^+ ^MLN cells (A) and were analyzed for expression of the markers CD80, CD86, CD40, OX40L, and CD11b at 2 hours (B) and 48 hours (C) post exposure. Marker expression is shown as median fluorescence intensity (MFI). Data shown represent MLN cells pooled from 3 animals per group and are representative of experiments performed twice.

The number of lung cells within the CD11c^+^CD11b^+ ^population (Figure 4A) was not significantly altered following NO_2 _exposure (Figure 4B). These cells were on average 81% ± 2.6% MHCII^+ ^regardless of air or NO_2 _treatment or time point (data not shown). Despite already expressing high levels of MHCII, the expression level of the co-stimulatory molecules CD40 and CD86 did not change substantially following NO_2 _exposure (Figure 4C). In fact, less than 10% of the CD11c^+^CD11b^+ ^population in the lung expressed CD40 and CD86 (data not shown). On average, 8.7% ± 2.1% of the cells were F4/80^+^, or potential macrophages [21]. CD11c and CD11b are typically expressed by myeloid DCs, the subset of DCs believed to be exceptionally well-suited for antigen presentation and activation of naïve CD4^+ ^T cells to become T effector cells [18]. Additionally, we measured an increase in CD11b expression within the CD11c^+^MHCII^+ ^population in the MLN following NO_2 _exposure (Figure 5C), indicating that CD11c^+^CD11b^+ ^cells may be particularly important in trafficking to the lymph node and presenting antigen to T cells under pro-inflammatory conditions.

### Mature dendritic cells (CD11c^+^MHCII^+^) are present in the mediastinal lymph node (MLN) following NO_2 _exposure

The lung-draining mediastinal lymph nodes (MLNs) were pooled from 3 air/ova- or NO_2_/ova-exposed mice per group. Individual analysis of the BAL from each of these mice indicated they were all representative of their particular exposure group. In contrast to pulmonary macrophages, which are also CD11c^+^, pulmonary DCs migrate to draining lymph nodes upon maturation where they are able to stimulate naïve CD4^+ ^T cells [13]. 2 hours following exposure to 15 ppm NO_2 _and 3.4% ova, there was a slight increase in CD11c^+^MHCII^+ ^DCs within the MLN (3.71% to 4.17% of CD45^+ ^cells) (Figure 5A). At 2 hours, these CD11c^+^MHCII^+ ^cells displayed markers indicative of maturation (Figure 5B). However, by 48 hours post exposure to NO_2 _and ova, MLN DCs displayed dramatic upregulation of the co-stimulatory molecules CD80, CD86, and OX40L, consistent with maturation and a phenotype capable of inducing a T cell-mediated inflammatory response [16,29] (Figure 5C). Furthermore, infiltrating MLN DCs were increasingly CD11b^+ ^at 48 hours, consistent with the phenotype of a myeloid or inflammatory DC.

### NO_2_-exposed pulmonary CD11c^+ ^cells produce pro-inflammatory cytokines and induce T cell cytokine production ex vivo

**Figure 6:**
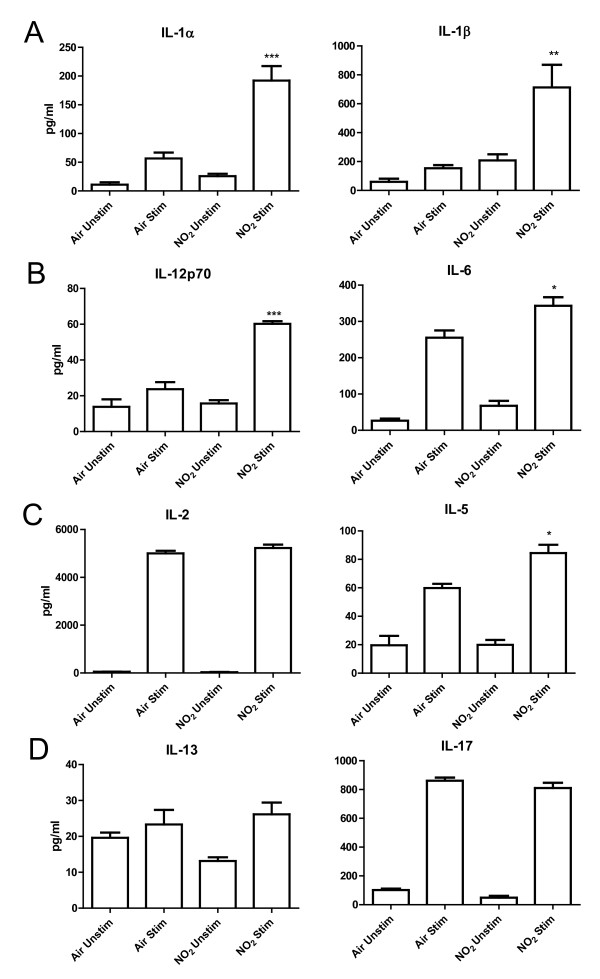
**Effects of NO**_**2 **_**inhalation on antigen capture in CD11c**^**+**^**CD11b**^**+ **^**lung cells *in vivo*. **Mice were exposed to air or 15 ppm NO_2 _for one hour followed by oropharyngeal aspiration of 50 μg of ova-Alexa 647. Lungs were harvested 2 and 48 hours later, single cell suspensions were generated and stained with antibodies, and cells were analyzed by flow cytometry. Dead cells were excluded by FSC and SSC gating and then total lung cells were gated comparing CD11c and CD11b expression, with future analyses focusing on the CD11c^+^CD11b^+ ^population (bold gate, A). The percent of CD11c^+^CD11b^+ ^cells within the lung was determined (B) and uptake of the antigen ova-Alexa 647 was quantified by median fluorescence intensity (MFI) (C). NO_2_-exposed animals are represented by a thick line while air-exposed animals are represented by a thin line. Grey, filled histograms are naive controls (did not receive ova-Alexa 647) (C). Data shown are mean ± SEM with 4 animals per group and are representative of experiments performed twice. * denotes *p *< 0.05 and ** denotes *p *< 0.01 versus air control at the same time point by unpaired Student's t test. ^ denotes *p *< 0.05 and ^^ denotes *p *< 0.01 versus 2 hours post NO_2 _exposure by unpaired Student's t test.

**Figure 7:**
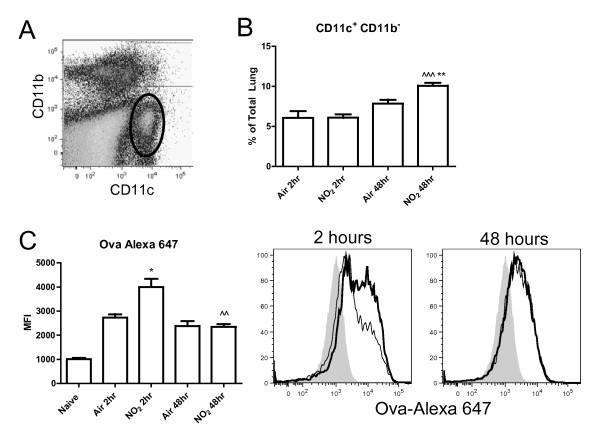
**Pro-inflammatory cytokine production and induction of CD4**^**+ **^**T cell cytokine production *ex vivo *by NO**_**2**_**-exposed pulmonary CD11c**^**+ **^**cells**. Mice were exposed to air or 15 ppm NO_2 _for 1 hour followed by 30 minutes of aerosolized 3.4% ova. Forty-eight hours later, CD11c^+ ^cells were purified from lungs via positive selection and used as antigen presenting cells co-cultured with CD4^+ ^T cells purified from the spleen of OTII transgenic mice via positive selection. CD11c^+ ^cells (2 × 10^6 ^cells/ml) co-cultured with T cells (1 × 10^6 ^cells/ml) were stimulated with 100 μg/ml ova_323-339 _and cultured for 4 days or left unstimulated in media alone. Conditioned medium were analyzed via BioPlex for pro-inflammatory mediators associated with CD11c^+ ^cell activation (IL-1α and IL-1β) (A) and T cell polarization (IL-12p70 and IL-6) (B). Cytokines associated with T cell proliferation (IL-2) (C) as well as cytokines produced by activated Th2 cells (IL-5 and IL-13) (C, D) and Th17 cells (IL-17) (D) were measured. Data shown are mean ± SEM with 6 mice per group. * denotes *p *< 0.05, ** denotes p < 0.01, *** denotes p < 0.001 compared with Air Stim as determined by one-way ANOVA with Bonferroni post test.

**Figure 8:**
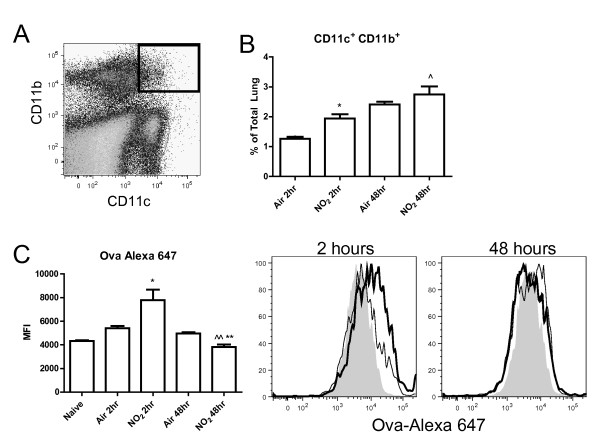
**Effects of NO**_**2 **_**inhalation on antigen capture in CD11c**^**+**^**CD11b**^- ^**lung cells *in vivo***. Mice were exposed to air or 15 ppm NO_2 _for one hour followed by oropharyngeal aspiration of 50 μg of ova-Alexa 647. Lungs were harvested 2 and 48 hours later, single cell suspensions were generated and stained with antibodies, and cells were analyzed by flow cytometry. Dead cells were excluded by FSC and SSC gating and then total lung cells were gated comparing CD11c and CD11b expression, with future analyses focusing on the CD11c^+^CD11b^- ^population (bold gate, A). The percent of CD11c^+^CD11b^- ^cells within the lung was determined (B) and uptake of the antigen ova-Alexa 647 was quantified by median fluorescence intensity (MFI) (C). NO_2_-exposed animals are represented by a thick line while air-exposed animals are represented by a thin line. Grey, filled histograms are naive controls (did not receive ova-Alexa 647) (C). Data shown are mean ± SEM with 4 animals per group and are representative of experiments performed twice. * denotes *p *< 0.05 and ** denotes *p *< 0.01 versus air control at the same time point by unpaired Student's t test. ^^ denotes *p *< 0.01 and ^^^ denotes *p *< 0.001 versus 2 hours post NO_2 _exposure by unpaired Student's t test.

Mature DCs have been documented to possess increased antigen presentation capabilities, strongly inducing T cell proliferation and cytokine production [16]. Therefore, we investigated whether CD11c^+ ^pulmonary cells from NO_2_-exposed animals have increased pro-inflammatory and T cell activating properties compared to those from air-exposed mice. Mice were exposed to either air or 15 ppm NO_2 _for one hour followed by 30 minutes of 3.4% ova. We then waited 48 hours as we have previously reported that separating the time between NO_2 _and the initial ova exposure increases sensitization to ova [6]. Thus, 48 hours following NO_2 _and ova exposure, CD11c^+ ^cells were removed from total lung digestions by positive selection and co-cultured with naïve splenic CD4^+ ^transgenic OTII T cells, which possess T cell receptors specific only for the antigenic peptide of ovalbumin, ova_323-339 _[39]. Since the majority of the immunostimulatory CD11c^+ ^cells that had engulfed the ovalbumin encountered during the inhaled exposure were likely no longer in the lung (see figures 6C and 7C), the cells were then either cultured *in vitro *with 100 μg of ova_323-339 _(stimulated) or in media alone (unstimulated). The cells were co-cultured for 96 hours and the supernatants were then analyzed for an array of cytokines and chemokines associated with DC activation, T cell activation, and Th2 polarization. When comparing stimulated cells, the CD11c^+ ^cells from NO_2_-exposed animals produced significant amounts of IL-1α and IL-1β, a Th17-polarizing cytokine [34], versus air-exposed controls (Figure 8A). NO_2_-exposed CD11c^+ ^cells also secreted increased quantities of IL-12, a Th1 polarizing cytokine, and IL-6, a Th2 polarizing cytokine [30,31](Figure 8B). Interestingly, air-exposed and NO_2_-exposed CD11c^+ ^cells were equally capable of promoting T cell proliferation, as measured by IL-2 production (Figure 8C). However, the T cells co-cultured with NO_2_-exposed CD11c^+ ^cells produced significantly larger amounts of IL-5 (Figure 8C), with no difference in IL-13 or IL-17 (Figure 8D).

### NO_2 _induces pulmonary CD11c^+ ^cells to capture antigen and travel to the mediastinal lymph node

DCs are particularly well-equipped for activating T cells as they are capable of capturing and processing antigens, travelling to the draining lymph node, and presenting those antigens to T cells [16,18]. Under inflammatory conditions, DCs increase in rate of turnover and total numbers within the lung and in transit to the draining lymph node [44,45]. To further study DC trafficking and maturation following NO_2 _exposure, Alexa 647-labeled ova was employed [21]. To test if conventional CD11c^+ ^DCs increase antigen uptake following NO_2 _exposure, C57BL/6 mice were exposed to 1 hour of 15 ppm NO_2 _or air followed by oropharyngeal aspiration of ova-Alexa 647. We focused on cells expressing CD11c as these populations displayed maturation following NO_2 _exposure in previous studies (Figure 3). We separately analyzed CD11c^+^CD11b^- ^and CD11c^+^CD11b^+ ^cells, as before, to identify differences between these two populations that contain DCs (Figure 6A). Two hours post-exposure, pulmonary CD11c^+^CD11b^- ^cells contained increased amounts of ova-Alexa 647 in the NO_2_- versus air-exposed mice (Figure 6C). At 48hours, ova-Alexa 647^+ ^CD11c^+^CD11b^- ^cells were still present in both the air- and NO_2_-treated groups with equivalent retention of labeled ova per cell (Figure 6C) despite the increased percentage of CD11c^+^CD11b^- ^cells in the lungs of NO_2_-exposed animals (Figure 6B).

**Figure 9:**
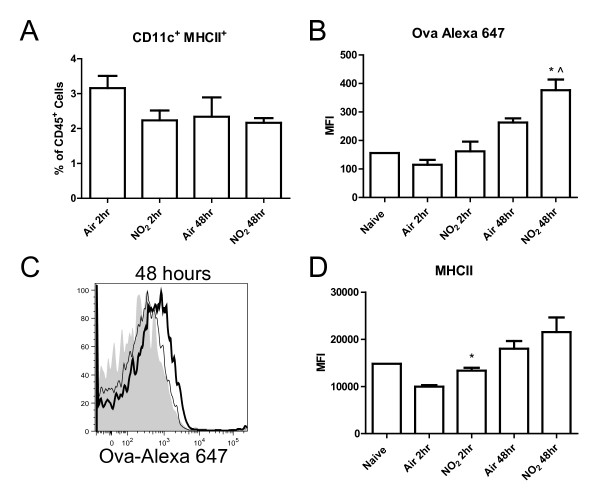
**Effects of NO**_**2 **_**inhalation on antigen-containing dendritic cells in the mediastinal lymph node**. Mice were exposed to air or 15 ppm NO_2 _for 1 hour followed by oropharyngeal aspiration of 50 μg of ova-Alexa 647. MLN were harvested 2 and 48 hours later, single cell suspensions were generated and stained with antibodies, and cells were analyzed by flow cytometry. CD11c^+^MHCII^+ ^cells (DCs) were gated as a percentage of CD45^+ ^cells in the MLN (A). DC retention of the antigen ova-Alexa 647 was quantified by the median fluorescence intensity (MFI) of Alexa 647 (B). The histogram represents ova-Alexa 647 retention at 48 hours post exposure (C). NO_2_-exposed animals are represented by a thick line while air-exposed animals are represented by a thin line. Grey, filled histograms are naive controls (did not receive ova-Alexa 647) (C). The maturation status of the DC population was assessed by expression of MHCII (D). Data shown are mean ± SEM with 4 animals per group and are representative of experiments performed twice. * denotes *p *< 0.05 versus air control at the same time point by unpaired Student's t test. ^ denotes *p *< 0.05 versus 2 hours post NO_2 _exposure by unpaired Student's t test.

Furthermore, while the total cell numbers from the lung were unchanged by NO_2_/ova, CD11c^+^CD11b^+ ^cells increased as a percentage of total lung cells 2 hours post NO_2_/ova-Alexa 647 exposure versus air control and elevated further by 48 hours (Figure 7B). NO_2 _exposure induced CD11c^+^CD11b^+ ^cells to increase antigen capture, as shown by a shift in the median fluorescence intensity of ova-Alexa 647 at 2 hours compared with the air control (Figure 7C). These CD11c^+^CD11b^+^ova-Alexa 647^+ ^cells were diminished at 48 hours versus the air control, potentially due to migration of this population to the MLN (Figure 7C, 9B, C). As shown previously, CD11c^+^CD11b^+ ^cells from both air- and NO_2_-exposed animals expressed high levels of MHCII (Figure 4C), but the levels of MHCII were significantly higher on CD11c^+^MHCII^+ ^cells from NO_2_-exposed MLNs at 2 hours (Figure 9D). By 48 hours, CD11c^+^MHCII^+ ^cells (DCs) in the MLN contained increased amounts of ova-Alexa 647 in NO_2_- versus air-exposed animals (Figure 9B, C), indicating that NO_2 _exposure promoted antigen capture and DC transit to the draining lymph node.

## Discussion

The incidence of allergic asthma continues to rise throughout industrialized countries and nations in transition [1], where the concentrations of air pollutants, such as NO_2_, are also increasing [46,47]. Studies have correlated an increased likelihood of developing asthma [12] and worsening asthma symptoms [10] with high levels of ambient NO_2_. Respiratory viral infections also result in NO_2 _production within the lung [48,49] and data suggest that certain severe viral infections early in life increase the risk of developing asthma [48]. In our previous studies, we have demonstrated that in mice, NO_2 _can act as an adjuvant, promoting allergic sensitization to an innocuous inhaled antigen [6]. Therefore, understanding the mechanisms by which NO_2 _promotes inappropriate adaptive immune responses will provide powerful insight concerning the effects of pollution and viral infection on respiratory health, the development of asthma, the balance between inhalational tolerance and allergy, and potentially provide mechanistic targets for prevention or treatment.

CD11c^+ ^DCs are critical in activating naïve CD4^+ ^T cells [16], the products of which are primarily responsible for the symptoms of allergic asthma [36,50]. Multiple inhaled environmental agents have adjuvant activities and lead to allergic sensitization, including endotoxin [51,52], triacylated lipopeptides [53], residual oil fly ash [54,55], diesel exhaust particles [56-58], cigarette smoke [59], ultrafine particles [60], and house dust mite [61,62]. Proposed mechanisms for this inappropriate immune response promoted by environmental agents include disruption of the epithelial barrier, leading to cytokine and chemokine production by epithelial cells, as well as antigen access to underlying DCs [25,63] and altered DC function [60,64-69]. Because CD11c^+ ^DCs are crucial for mounting naïve T cell responses, we hypothesized that eliminating CD11c^+ ^cells during NO_2_-promoted allergic sensitization would minimize features of allergic asthma in mice. To test this hypothesis, we utilized transgenic CD11c-DTR mice, in which temporary depletion of CD11c^+ ^DCs is possible.

To use the CD11c-DTR mice, we adjusted our model of NO_2_-promoted allergic sensitization to include only one sensitization. Our data demonstrate that even a single exposure to 15 ppm NO_2 _for 1 hour followed by 30 minutes of aerosolized ova immediately after is sufficient to induce allergic sensitization to ova. Interestingly, we measured substantial increases in IL-4, IL-5, IL-13, and IL-17 from restimulated CD4^+ ^T cells as well as substantial increases in eosinophilia and neutrophilia in the BAL, indicating that our one sensitization model polarizes CD4^+ ^T cells down both Th2 and Th17 pathways. These pathways overlap as the mediator IL-6 is important in promoting both pathways [30-32] and was secreted at elevated levels by DCs from NO_2_-exposed mice. Systemic administration of DT to CD11c-DTR transgenic mice prior to sensitization resulted in a decrease in the total white blood cell count and eosinophilia in the BAL of Tg^+ ^animals versus Tg^- ^animals. Similar differences were measured for cytokine production from *in vitro *restimulated CD4^+ ^T cells. Thus, systemic depletion of CD11c^+ ^cells provides mechanistic insight into the important role of CD11c^+ ^cells in NO_2_-promoted allergic sensitization. However, our findings suggest that while CD11c^+ ^cells are important in NO_2_-promoted allergic sensitization, depleting these cells during sensitization does not abolish all parameters of the allergic asthma phenotype, as we were unable to measure a difference in the levels of ovalbumin-specific immunoglobulins from the serum of Tg^+ ^and Tg^- ^animals. This may be due, in part, to the low levels of antigen-specific immunoglobulins generated as a consequence of our modified sensitization regimen in which mice were only exposed to inhaled NO_2 _and ova on a single occasion compared to our previously-published model that involved two sensitizations [6]. Importantly, when DT was administered via i.p. injection, depletion of CD11c^+ ^cells within the lung occurred as expected in Tg^+ ^animals [17]. However, this depletion does leave some CD11c^+ ^cells present in the lung, inducing approximately an 80% decrease from 5.8% to 1% of total lung cells. Thus, it is possible that this depletion minimizes the initial number of naïve T cells activated, but that after repeated exposure to antigen, clonal expansion allows for the development of some of the features of the Th2 response, including the expansion of the eosinophil population, their recruitment to the airway, and the generation of antigen-specific immunoglobulins. Additionally, depleting CD11c^+ ^cells during sensitization may alter rather than reduce the immune response, changing the outcomes associated with sensitization. However, we did not measure any qualitative alterations in the type of immune response provoked in the CD11c-DTR Tg^+ ^mice.

It is important to consider other CD11c^+ ^cell populations in addition to mDCs that are also affected by DT administration to Tg^+ ^animals. Plasmacytoid DCs (pDCs) and alveolar macrophages also express CD11c, albeit to a lesser extent than mDCs [21]. Plasmacytoid DCs promote tolerance by decreasing the capability of myeloid DCs to induce T cell proliferation as well as promoting CD4^+ ^T regulatory cell proliferation [26]. Thus, by depleting CD11c^+ ^cells within the lung, depletion of both pro-inflammatory mDCs as well as anti-inflammatory pDCs occurs. Despite this caveat, CD11c-DTR mice have revealed the importance of CD11c^+ ^cells in promoting allergic sensitization in other models.

Our current studies demonstrate that one mechanism by which NO_2 _may act as an adjuvant is through the activation and recruitment of pulmonary CD11c^+ ^DCs, a cell type believed to act as the bridge between innate and adaptive immunity [25]. We measured increased numbers of CD11c^+^CD11b^- ^cells and minimal changes in CD11c^+^CD11b^+ ^cells in the lungs 2 hours following NO_2 _exposure, all of which have the potential to be DCs. Other adjuvants have been shown to work similarly, including the well known adjuvant alum, which causes the recruitment of inflammatory DCs to the site of injection (peritoneum) and subsequently to the draining lymph nodes [22]. Additionally, the CD11c^+^CD11b^- ^population in the lung significantly upregulated expression of MHCII, CD40, and OX40L 2 hours post-NO_2_, thereby enhancing its capability for antigen presentation and providing co-stimulation to promote Th2 responses. Myeloid DCs pulsed with ova and administered directly into mouse lungs also result in allergic sensitization to ova, indicating that these cells have the capability to induce T cell responses [24]. Furthermore, our data demonstrate that inhalation of 15 ppm NO_2 _for just 1 hour is sufficient to induce the production of MCP-1 within the lung, a chemokine associated with DC recruitment [70]. IL-6 is also increased in the BAL fluid 2 hours following NO_2 _exposure, which can induce a Th2 polarized environment [31].

From NO_2_-exposed mice, we measured increased protein in the BAL, indicative of cellular damage that may promote the activation of epithelial cells capable of secreting factors such as IL-6 [71] and other pro-inflammatory mediators [25]. Agents that cause damage to the lung epithelium, such as the proteases produced by house dust mites, have been shown to promote allergic sensitization through allowing access of the antigens to resident antigen presenting cells and inducing cytokine and chemokine production from epithelial cells [63]. Previous findings have demonstrated that 10 ppm NO_2 _induces the release of intracellular molecules such as HSP70 [6], which can act as an endogenous danger signal [72] and activate the transcription factor NF-κB, a key modulator in both innate and adaptive immune responses. Interestingly, NF-κB is capable of inducing IL-6 and MCP-1 gene expression [73] and is activated following NO_2 _exposure [6], providing a potential mechanism by which NO_2 _promotes CD11c^+ ^pulmonary cell activation and Th2 polarization. Our *in vitro *co-culture data indicate than an effect of NO_2 _solely on lung DCs is insufficient to explain the creation of a Th2/Th17-polarizing environment in the lung. *In vivo*, additional cell types, including epithelium, contribute to the effects of NO_2 _inhalation on CD4^+ ^T cell polarization. We have recently reported that NF-κB activation in the airway epithelium, as is also induced by NO_2 _exposure [6,74], is sufficient to activate mediastinal lymph node DCs and to promote a mixed Th2/Th17 response to an innocuous inhaled antigen [75].

Allergic asthma is primarily dependent upon the activities of CD4^+ ^T cells [2]. CD11c^+ ^DCs regulate the initiation of naïve CD4^+ ^T cell responses in multiple ways, including carrying antigens to the draining lymph node, processing and presenting these antigens, providing co-stimulation, and secreting cytokines [16]. In our studies, CD11c^+ ^pulmonary cells secreted increased amounts of IL-1α, IL-1β, IL-12p70 and IL-6 *ex vivo *following NO_2 _exposure, demonstrating that these cells alone produce pro-inflammatory mediators. When co-cultured with CD4^+ ^OTII cells, NO_2_-exposed pulmonary CD11c^+ ^cells promoted the production of IL-5 from OTII cells more so than air-exposed controls, implicating that NO_2_-exposed CD11c^+ ^cells induced increased Th2 cell activity. Although we measured increases in Th1- (IL-12p70), Th2- (IL-6), and Th17-(IL-1) polarizing cytokines, it is important to remember that these co-cultures did not include epithelial cells. It is likely that epithelial cells also influence the outcome of DC and CD4^+ ^T cell interactions, as epithelial cells are capable of secreting multiple mediators that affect T cell polarization, promoting the development of a Th2 immune response [25]. This may also be one of the reasons why we observed no differences in the secretion of IL-17 by the CD4^+ ^T cells co-cultured with DCs from NO_2_-exposed mice compared to those from the air-exposed mice.

NO_2 _exposure also caused pulmonary CD11c^+^CD11b^- ^and CD11c^+^CD11b^+ ^cells to increase antigen capture and trafficking to the draining lymph node, increasing the opportunity for a T cell response. Interestingly, 2 hours following NO_2_, both CD11c^+^CD11b^- ^and CD11c^+^CD11b^+ ^cells within the lung contained increased amounts of ova-Alexa 647, but by 48 hours contained equal (CD11c^+^CD11b^-^) or lower (CD11c^+^CD11b^+^) amounts of ova-Alexa 647 than air controls. Also at 48 hours, CD11c^+^MHCII^+ ^cells containing ova-Alexa 647 increased in the MLN, indicating that NO_2 _inhalation induced trafficking of these cells to the MLN.

## Conclusions

Collectively, the results of the studies presented herein demonstrate that CD11c^+ ^cells, which include conventional DCs, are critical in NO_2_-promoted allergic sensitization, as depletion of these cells only during sensitization diminished multiple features of allergic asthma in mice. NO_2 _inhalation significantly impacts pulmonary CD11c^+ ^cells, as shown by increased cytokine production, upregulation of maturation markers, increased antigen uptake, and improved ability to stimulate naïve T cells, all of which occur in a temporally-coordinated manner. Future work will be required to identify critical mediators of these effects through the use of neutralizing antibodies and knockout mice, as well as by further refining the direct or indirect effects of NO_2 _on specific subsets of pulmonary DCs, including the CD103^+ ^population that expresses adherens junction proteins and interacts closely with the epithelial cells at the interface of the airway with the external environment [76]. Additionally, we currently cannot exclude the possibility that other factors affected by NO_2 _exposure, such as the pH of the airway surface lining fluid and oxidation/nitration events within proteins [77-79], may play a role in the adjuvant effects of NO_2_. Understanding the mechanisms underlying the inappropriate T cell activation induced following exposure to environmental pollutants or endogenously-generated oxidant gasses, such as NO_2_, will provide insight to allergic sensitization as it occurs in humans, providing further understanding of the causes of allergic asthma.

## List of Abbreviations

APC: antigen-presenting cell; BAL: bronchoalveolar lavage; CD: cluster of differentiation; DC: dendritic cell; DT: diphtheria toxin; DTR: diphtheria toxin receptor; IL: interleukin; MHCII: major histocompatability complex class 2; MLN: mediastinal lymph node; NO_2_: nitrogen dioxide; ova: ovalbumin; ppm: parts per million; Tg+: transgenic; Tg-: transgene negative; Th: T helper.

## Competing interests

The authors declare that they have no competing interests.

## Authors' contributions

SRH designed the experiments, executed the studies, and drafted the manuscript. JLA bred, genotyped, and maintained mice for the studies. SAP and JLA assisted with cell staining for flow cytometry. LAWL assisted with study design and cell staining. BTS and JEB assisted with analysis of flow cytomentry data. MEP conceived of the studies, coordinated their execution, and revised the manuscript. All authors read and approved the final manuscript.
